# Supplementary material for: SDM6A: A Web-Based Integrative Machine-Learning Framework for Predicting 6mA Sites in the Rice Genome
Source: Mol Ther Nucleic Acids. 2019 Aug 16;18:131–41. doi: 10.1016/j.omtn.2019.08.011 (PMC6796762; doi:10.1016/j.omtn.2019.08.011)
Supplement: Document S1. Figures S1 and S2 and Tables S1 and S2 [file mmc1.pdf]

OMTN, Volume *18*

Supplemental Information

SDM6A: A Web-Based Integrative Machine-Learning Framework for Predicting 6mA Sites in the Rice Genome

Shaherin Basith, Balachandran Manavalan, Tae Hwan Shin, and Gwang Lee

Figure S1. Performance of four different ML classifiers with respect to using five feature descriptors to distinguish between 6mA sites and non-6mA sites on independent dataset. (A) RF, (B) ERT, (C) XGB, and (D) SVM.

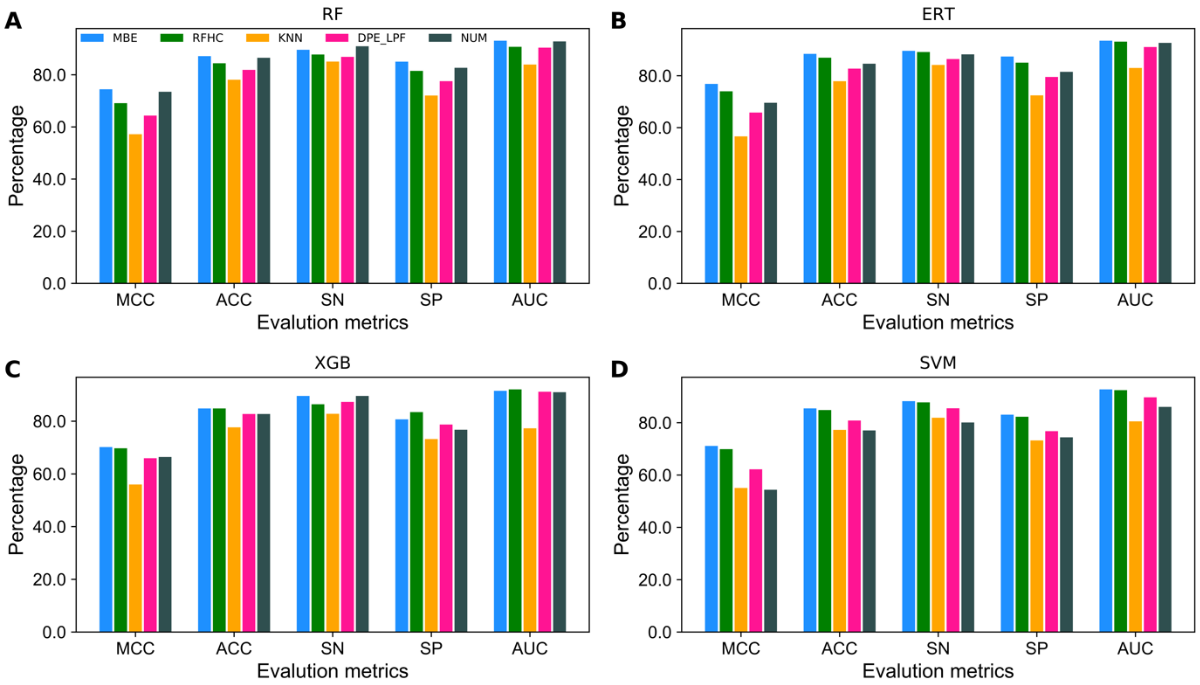

Figure S2. Performance of four different ML classifiers with respect to using nine feature descriptors to distinguish between 6mA sites and non-6mA sites on benchmark dataset. (A) RF, (B) ERT, (C) XGB, and (D) SVM.

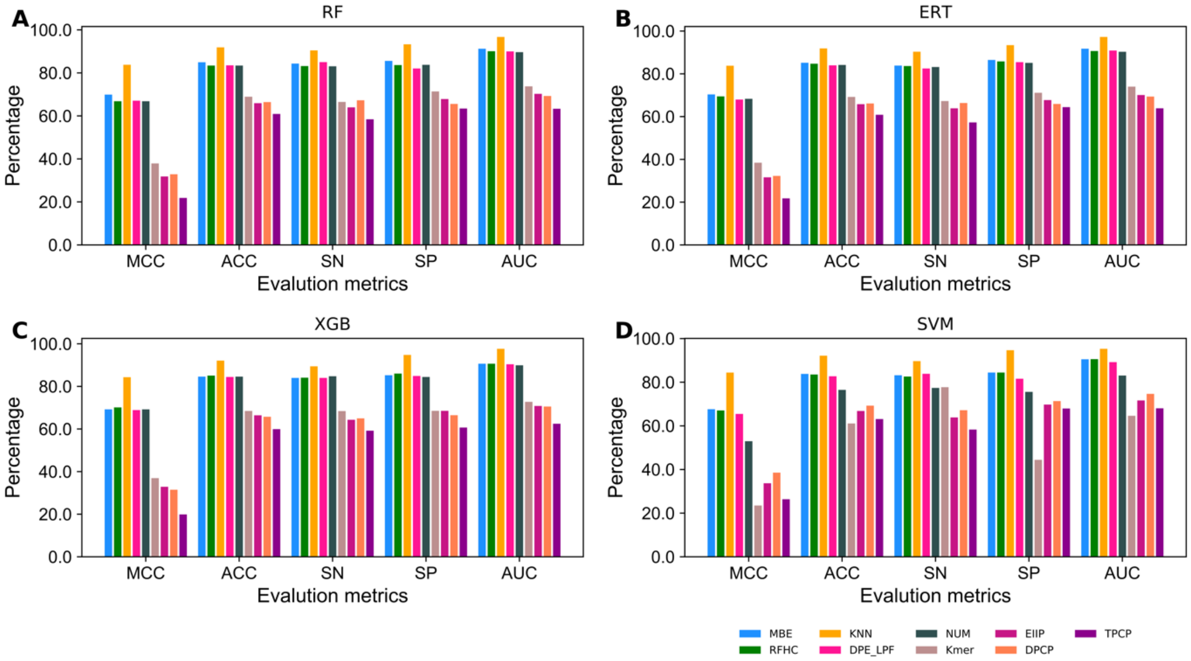

Table S1. Performances of four different classifiers using optimal feature set in distinguishing 6mAs and non-6mAs with respect to four different feature encodings.

Feature encoding	Method	MCC	ACC	SN	SP	AUC
MBE	RF	0.718	0.859	0.864	0.855	0.922
	ERT	0.719	0.860	0.858	0.861	0.925
	SVM	0.719	0.860	0.852	0.867	0.921
	XGB	0.705	0.852	0.867	0.838	0.921
NUM	RF	0.675	0.838	0.839	0.836	0.902
	ERT	0.686	0.843	0.836	0.850	0.910
	SVM	0.575	0.788	0.794	0.781	0.849
	XGB	0.701	0.851	0.854	0.847	0.914
RFHC	RF	0.687	0.843	0.831	0.856	0.909
	ERT	0.706	0.853	0.846	0.860	0.915
	SVM	0.717	0.858	0.838	0.878	0.917
	XGB	0.702	0.851	0.851	0.851	0.918
DPE_LPF	RF	0.684	0.842	0.843	0.841	0.912
	ERT	0.690	0.845	0.830	0.860	0.912
	SVM	0.691	0.846	0.850	0.841	0.911
	XGB	0.696	0.848	0.853	0.842	0.907

Table S2. Performance comparison between SDM6A and i6mA-Pred on benchmark dataset using jackknife test.

Method	MCC	ACC	SN	SP	AUC	P-value (AUC)
SDM6A	0.774	0.887	0.878	0.896	0.948	—
i6mA-Pred	0.660	0.831	0.830	0.831	0.886	<0.000001

First column represents the method name employed in this study. The second, third, fourth, and the fifth columns, respectively, represent the MCC, accuracy (ACC), sensitivity (SN), and specificity (SP). The sixth and the seventh columns, respectively, represent the AUC and pairwise comparison of ROC area under curves (AUCs) between SDM6A and the i6mA-Pred using a two-tailed *t*-test [1].

Reference

1. Hanley, JA, and McNeil, BJ (1982). The meaning and use of the area under a receiver operating characteristic (ROC) curve. *Radiology* **143**: 29-36.
