# Supplementary material for: SDM6A: A Web-Based Integrative Machine-Learning Framework for Predicting 6mA Sites in the Rice Genome
Source: Mol Ther Nucleic Acids. 2019 Aug 16;18:131–41. doi: 10.1016/j.omtn.2019.08.011 (PMC6796762; doi:10.1016/j.omtn.2019.08.011)
Supplement: Document S2. Article plus Supplemental Information [file mmc2.pdf]

Molecular Therapy
Nucleic Acids
Original Article

SDM6A: A Web-Based Integrative Machine-Learning Framework for Predicting 6mA Sites in the Rice Genome

Shaherin Basith,[1,2] Balachandran Manavalan,[1,2] Tae Hwan Shin,[1] and Gwang Lee[1]

[1]Department of Physiology, Ajou University School of Medicine, Suwon, Republic of Korea

DNA N^6-adenine methylation (6mA) is an epigenetic modification in prokaryotes and eukaryotes. Identifying 6mA sites in rice genome is important in rice epigenetics and breeding, but non-random distribution and biological functions of these sites remain unclear. Several machine-learning tools can identify 6mA sites but show limited prediction accuracy, which limits their usability in epigenetic research. Here, we developed a novel computational predictor, called the Sequence-based DNA N^6-methyladenine predictor (SDM6A), which is a two-layer ensemble approach for identifying 6mA sites in the rice genome. Unlike existing methods, which are based on single models with basic features, SDM6A explores various features, and five encoding methods were identified as appropriate for this problem. Subsequently, an optimal feature set was identified from encodings, and corresponding models were developed individually using support vector machine and extremely randomized tree. First, all five single models were integrated via ensemble approach to define the class for each classifier. Second, two classifiers were integrated to generate a final prediction. SDM6A achieved robust performance on cross-validation and independent evaluation, with average accuracy and Matthews correlation coefficient (MCC) of 88.2% and 0.764, respectively. Corresponding metrics were 4.7%–11.0% and 2.3%–5.5% higher than those of existing methods, respectively. A user-friendly, publicly accessible web server (http://thegleelab.org/SDM6A) was implemented to predict novel putative 6mA sites in rice genome.

INTRODUCTION

Recent breakthroughs in the fields of molecular biology and genomics have made it possible to determine the functional significance of DNA modifications. Dynamic DNA modifications, including methylation and demethylation, are major epigenetic mechanisms in the regulation of gene expression.[1] DNA methylations at the 5th position of the pyrimidine ring of cytosine (5-methylcytosine [5mC]) and at the 6th position of the purine ring of adenine (N^6-adenine methylation [6mA]; N^6-methyladenine) are the most common DNA modifications in eukaryotes and prokaryotes, respectively.[2] 5mC sites are well-known because they show widespread distribution and play multifaceted roles. However, 6mA sites have not been extensively investigated because of their non-uniform distribution across the genome. The distribution and function of 6mA modifications has been studied in unicellular eukaryotes; however, until recently, the nature of these alterations in multicellular eukaryotes was unclear.[3] Several new studies have shed light on the distribution and contrasting regulatory functions of 6mA modifications in multicellular eukaryotes, such as *Caenorhabditis elegans*, *Danio rerio*, *Drosophila melanogaster*, *Mus musculus*, *Tetrahymena*, and *Xenopus laevis*.[4–10]

Advancements in methodology used to detect 6mA sites have allowed several studies to demonstrate the biologically significant roles of 6mA sites in DNA replication and mismatch repair, transposable element activity, epigenetic inheritance, nucleoid segregation, and regulation of transcription in prokaryotic and eukaryotic genomes.[2,5,11,12] Experimental techniques for identifying 6mA sites include coupling immunoprecipitation with next-generation sequencing,[13] restriction enzyme-assisted sequencing with DpnI-assisted N^6-methyladenine sequencing,[14] single-molecule real-time (SMRT) sequencing,[15] capillary electrophoresis and laser-induced fluorescence (CE-LIF) based on fluorescence labeling of deoxyribonucleotides with 4,4-difluoro-5,7-dimethyl-4-bora-3a,4a-diaza-s-indacene-3-propionyl ethylenediamine (BODIPY FL EDA),[16] and DNA immunoprecipitation with 6mA-specific antibodies.[6] These methods, however, are typically labor-intensive and offer limited coverage of 6mA epigenetics. Advanced-profiling techniques have not been widely used in biological studies because of their prohibitively high costs and complexity. Nonetheless, the information these approaches can provide on 6mA sites is necessary for computational predictions.

Increasing numbers of novel DNA sequences and experimental complexities involved in detection of 6mA sites necessitate the development of new and efficient computational methods. Machine learning (ML) approaches are used to automate analytical model building for

Received 12 June 2019; accepted 8 August 2019;
https://doi.org/10.1016/j.omtn.2019.08.011.

[2]These authors contributed equally to this work.

Correspondence: Gwang Lee, Department of Physiology, Ajou University School of Medicine, Suwon, Republic of Korea.
E-mail: glee@ajou.ac.kr

This is an open access article under the CC BY license (http://creativecommons.org/licenses/by/4.0/).

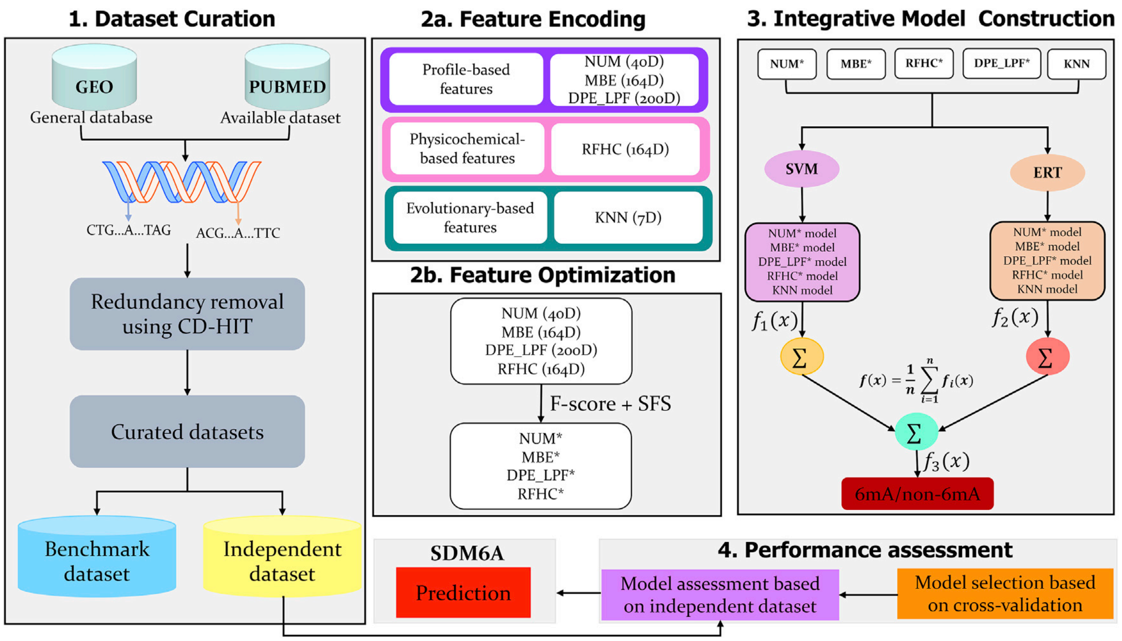

Figure 1. Overall Framework of SDM6A

The four major steps include: (1) data collection and pre-processing, (2) feature extraction and optimization using two-step feature selection protocol, (3) parameter optimization and construction of ensemble model, and (4) performance assessment and web server development.

rapid and accurate outcome predictions. Zhou et al. used mass spectrometry, immunoprecipitation, and sequencing to examine the 6mA profile of rice (*Oryza sativa*) genome.[17] The information obtained in that study allowed for the development of three ML-based methods within a few months. i6mA-Pred, the first ML-based computational method for identifying 6mA sites in the rice genome, was developed by Chen et al.[18] i6mA-Pred is a support vector machine (SVM)-based method in which nucleotide (NT) chemical properties and frequency are used as features for encoding DNA sequences. Chen et al.[18] evaluated their proposed models using jack-knife cross-validation and obtained an accuracy of 83.13%. This method has been made publicly available in the form of an online web server.

Another group used a deep learning (DL) approach to identify 6mA sites via a convolution neural network; these findings are also publicly available on a web server. The proposed computational model, iDNA6mA, obtained the accuracy and Matthews correlation coefficient (MCC) of 86.64% and 0.732, respectively.[19] During the course of our study, Le[20] developed an SVM-based method using a continuous bag of nucleobases via Chou's 5-step rule.[19] Those models were evaluated using jack-knife cross-validation and showed an accuracy and MCC of 87.78% and 0.756, respectively. This method is not publicly available, and, therefore, could not be fully utilized as a rationale for our present study. Although these techniques have demonstrated good performance, they are not easily generalizable or transferable. Therefore, it is still necessary to develop an effective predictor for accurate identification of 6mA sites in the rice genome.

The sequence-based DNA N^6-methyladenine predictor (SDM6A), which is a two-layer ensemble learning-based predictor for correctly identifying 6mA sites in the rice genome (Figure 1), was developed to address the challenges and limitations present in existing methods. By exploring nine different feature encodings and four different classifiers, five different encodings (ring-function-hydrogen-chemical [RFHC] properties, numerical representation of nucleotides [NUM], mono-nucleotide binary encoding [MBE], a combination of dinucleotide binary encoding and local position-specific dinucleotide frequency [DPE_LPF], and K-nearest neighbor [KNN]) and two classifiers (SVM and extremely randomized tree [ERT]) were identified. Then, an optimal feature set was identified from the four encodings and KNN encoding used as such, whose corresponding models were developed independently using SVM and ERT classifiers. In the first layer, the five single models were integrated using an ensemble approach to define a class for each classifier. In the second layer, SVM and ERT were integrated to develop a final prediction model. Further validation of SDM6A was performed using our constructed independent dataset. Our results show that the proposed model outperformed previous state-of-the-art methods with higher prediction accuracy. We also provided a user-friendly online web server called SDM6A (http://thegleelab.org/SDM6A), which can be used as a preliminary screening tool for the detection of potential 6mA sites in the rice genome. This server will allow for effective screening of 6mA sites in the rice genome, thereby expediting and facilitating future plant breeding and genome research.

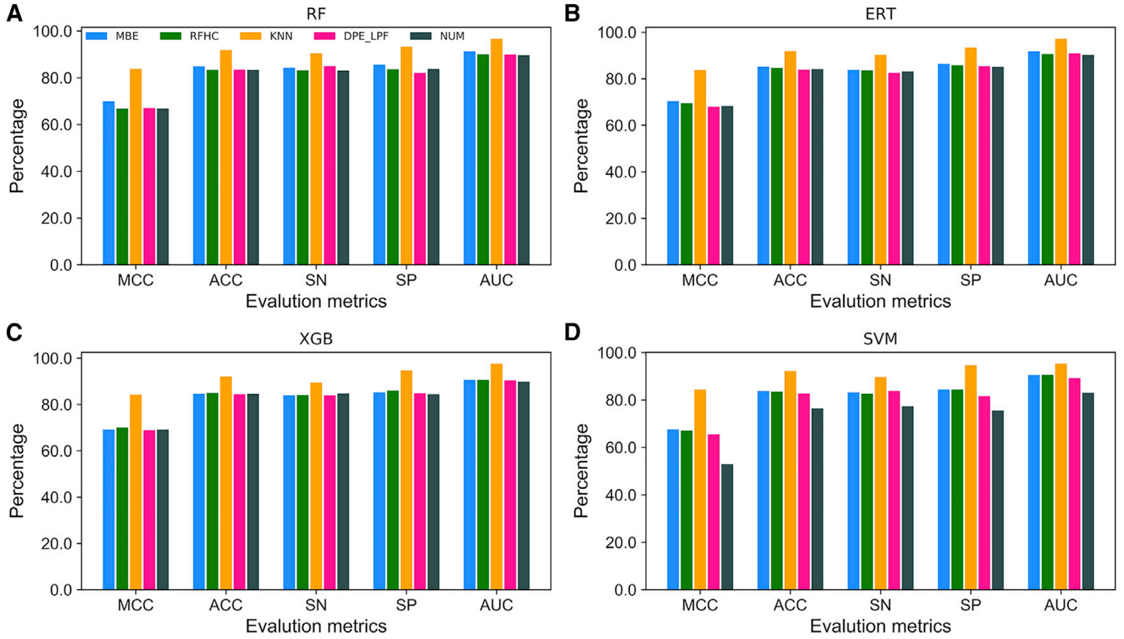

Figure 2. Performance of Four Different ML Classifiers with Respect to Using Five Feature Encodings to Distinguish between 6mA Sites and non-6mA Sites
(A) RF, (B) ERT, (C) XGB, and (D) SVM.

RESULTS AND DISCUSSION

Evaluating the Performance and Robustness of Different Feature Encodings

We evaluated the performance of five different feature encodings (categorized into three groups) using four different ML classifiers (random forest [RF], ERT, SVM, and extreme gradient boosting [XGB]). For each feature encoding method, an ML classifier was trained using 10-fold cross-validation (CV) with optimally tuned parameters based on a benchmark dataset. Figure 2 shows that KNN feature encoding achieved the best performance and outperformed other encodings for all four ML classifiers. However, the remaining four encodings (MBE, RFHC, NUM, and DPE_LPF) achieved similar performances for the three classifiers (RF, XGB, and ERT). Performances of the four encodings varied in the case of SVM.

The main objective of this study was to develop a robust predictor; therefore, 20 prediction models (5 feature encodings × 4 ML classifiers) were evaluated on an independent dataset to determine the transferability (robustness) of 10-fold CV performance. Figure S1 shows that KNN feature encoding achieved the lowest performance among the four ML classifiers, which is contrary to the results of 10-fold CV. The difference in accuracy (ΔACC) between 10-fold CV and independent evaluation was computed to summarize the robustness of each model. Figure 3 shows that KNN encoding underperformed mainly in terms of robustness (ΔACC ∼14%) for all four classifiers. Interestingly, XGB using MBE (84.6%) and RFHC (85.06%) encodings, and ERT (84.15%) and SVM (76.5%) using NUM encoding, showed robustness, with $\Delta ACC < 1.0$; however, the corresponding accuracies were unsatisfactory. The remaining 12 prediction models also underperformed slightly in terms of robustness, with $\Delta ACC < 2.0$. Overall, these results show that using different feature encodings or different classifiers could not generate a robust and highly accurate predictive model.

In addition to the above five feature encodings, four other encoding methods were explored including *K*mer (a linear combination of mono-, di-, tri-, tetra-, and penta-NT composition, encoded as a vector containing 1,364 elements), electron-ion interaction pseudo potential (PseEIIP), dinucleotide physicochemical properties (DPCP), and trinucleotide physicochemical properties (TPCP); these have been successfully used in previous studies.[21,22] Figure S2 shows that these four feature encodings achieved a lower performance, with the average accuracies ∼15%–23% lower than those of the five feature encodings discussed earlier, irrespective of ML classifiers used. Although these four feature encodings contributed in a significant manner previously, including 4mC site prediction,[21–23] they did not play any significant role in 6mA site prediction. Therefore, we excluded these four encodings from the subsequent analysis.

Determining Optimal Features for Four Feature Encodings

Even when 10-fold CV performances for the four classifiers are satisfactory, the original feature set may contain redundant features. Therefore, it is necessary to choose an optimal feature set for the construction of an efficient predictive model. In this study, a two-step feature optimization strategy (as described in the Materials and Methods) was used with respect to the four feature encodings. The KNN feature encoding was excluded from feature optimization

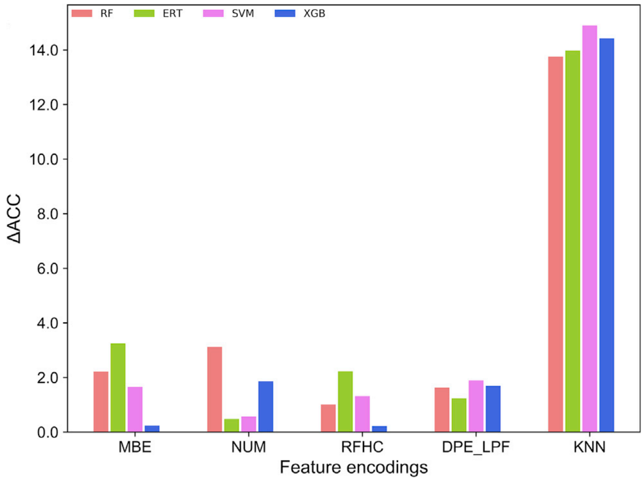

Figure 3. Absolute Differences in Accuracy (ΔACC) Computed between the Accuracy Obtained from 10-fold Cross-Validation and Independent Evaluation for Each Classifier with Respect to Different Encodings

because of its small feature dimension (7-dimension). Figure 4 shows ACC curves with gradual addition of features from the ranked feature list for the four classifiers based on four different encodings. For the three feature encodings (MBE, DPE_LPF, and RFHC), the ACC curve gradually improved and reached its maximum point, followed by a plateau upon addition of ranked features. Conversely, the NUM encoding rapidly reached maximal accuracy, which then declined. Here, a feature set that produced the highest accuracy was considered the optimal feature set. The best performance achieved by the four different classifiers with respect to the optimal feature set is shown in Table S1.

To validate whether feature optimization strategy improved predictive performance, the performances of optimal features (after feature optimization) were compared with those of the original features (before optimization). The results showed that all four methods using corresponding optimal features consistently improved in their respective performances (Figure 5A). However, the percentage of improvements varied among the methods. The average performance of SVM, RF, ERT, and XGB improved by 2.13%, 0.73%, 0.51%, and 0.4%, respectively, compared with their respective performances using original features. Moreover, optimal feature dimension was significantly reduced compared with that using original features. However, optimal feature dimension varied among the methods. The SVM, ERT, RF, and XGB optimal features contained 42.9%, 42.2%, 53.2%, and 63.1% of the original features, respectively (Figure 5B). These results demonstrate that feature optimization can effectively reduce feature dimension, thereby contributing to improved progressive performance.

Assessing Models Constructed Using Ensemble Strategy

In principle, the ensemble learning strategy can significantly improve model performance and generalizability compared with those of models trained using single-feature encoding or a combined set of features.[24–26] In the present study, five single feature-based models were integrated using an ensemble learning strategy. The predicted probability scores of five single feature-based models were summed up with different weights, and a default cut-off threshold of 0.5 was used to define the class for each classifier. Notably, the sum of five different weights was one, for which optimal values were determined using a grid search. As shown in Table 1, the classifiers RF, ERT, SVM, and XGB achieved similar performances; however, gaps between sensitivity (SN) and specificity (SP) varied among these four methods. Instead of selecting a final prediction model from Table 1, ensemble models were generated by exploring all possible combinations of four individual ML-based models. Conventionally, the predicted probability scores of two or more methods are averaged with equal weights; then, the average score is optimized to define class. Table 1 showed that an ensemble model (a combination of SVM and ERT, which is indicated as {2, 3} in Table 1) achieved the best performance, with MCC and ACC of 0.763 and 0.881, respectively. Specifically, MCC and ACC were 0.3%–1.1% and 0.1%–0.7% higher than those obtained using other methods developed in this study, which indicates marginal gains.

The performance of our best method ({2, 3}) was comparable to those achieved with state-of-the-art predictors, including i6mA-Pred and iDNA6mA. Specifically, the existing methods were trained and validated (k-fold CV) on the same benchmark dataset as that used in this study. Comparison with the best existing predictor, iDNA6mA, showed that ACC and MCC of our best-performing method were 1.4% and 3.01% higher, respectively. Notably, i6mA-Pred reported two predictive results, which were based on 10-fold CV and jackknife test.[18] In Table 1, we compared i6mA-Pred 10-fold CV results with our models. To compare i6mA-Pred jackknife result with our best model SDM6A ({2, 3}), we reconstructed our best model using jackknife test. According to the p value threshold of 0.05, our best model significantly outperformed i6mA-Pred (Table S2). Overall, the improved performance of the predictor developed in this study indicates that it was more accurate than other state-of-the-art predictors in distinguishing 6mA sites from non-6mA sites.

Performance Evaluation Using an Independent Dataset

Previously, several studies have proposed prediction models without any external evaluations.[27–31] However, when objectively evaluated using an independent dataset, these methods may not achieve the same performance as that using a benchmark dataset. In this study, we observed that KNN feature encoding achieved the best performance on a benchmark dataset but failed significantly on an independent evaluation. This further emphasizes the necessity of using an independent dataset to assess the robustness of the developed model.

Performance of Single ML-Based and Ensemble Models

All the models listed in Table 1 were evaluated using an independent dataset. Table 2 shows that the majority of the models (eleven) demonstrated an inconsistent performance when assessed using independent and benchmark datasets. The remaining SVM and three

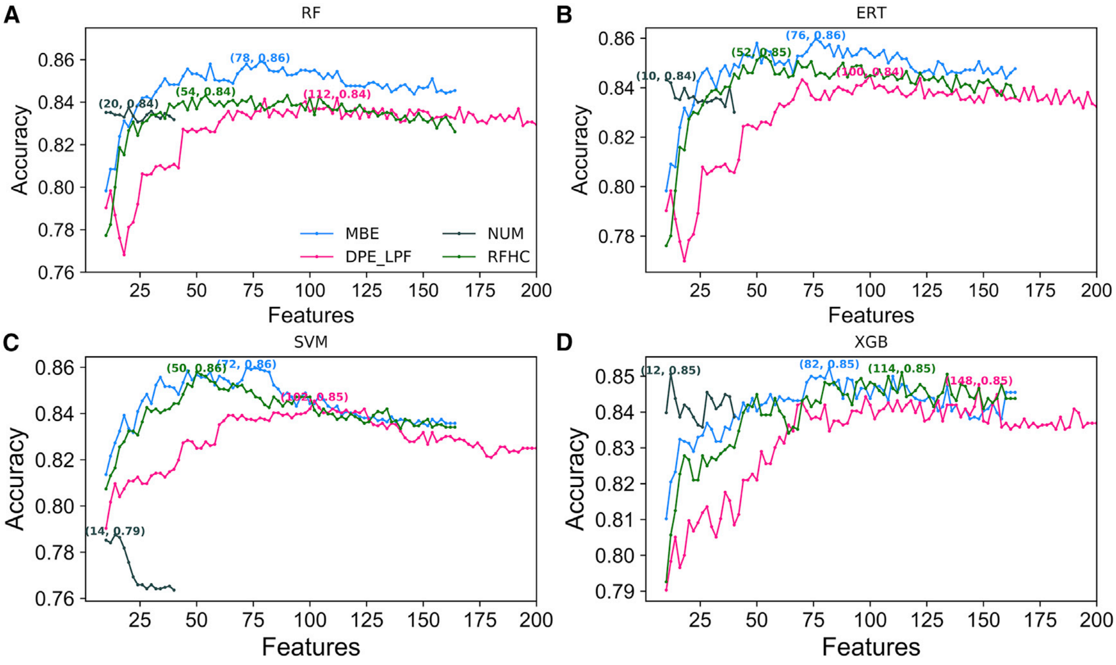

Figure 4. Sequential Forward Search for Discriminating between 6mA Sites and Non-6mA Sites

The x axis corresponds to the feature dimension and y axis represents its performance in terms of accuracy. The maximum accuracy obtained via 10-fold cross-validation is shown for each feature encoding. (A) RF, (B) ERT, (C) SVM, and (D) XGB.

ensemble models {2, 3}, {1, 2, 3}, and {1, 3, 4} achieved a consistent performance, with ΔACC < 0.5%. Of these, {2, 3} achieved the best performance, with MCC and ACC of 0.765 and 0.882, respectively. The MCC and ACC achieved by {2, 3} were 0.8%–5.0% and 0.4%–2.4% higher than those of the other models used in this study. Importantly, {2, 3} achieved the best and most reliable performance when assessed using both benchmark and independent datasets. This result shows that it is important to leverage different types of DNA characteristics using varied aspects; these characteristics can then be integrated, via an ensemble approach, into a unified computational framework, which generates a robust and improved predictor. The {2, 3} model selected in this study was designated as "SDM6A."

Comparing the Performance of SDM6A with That of the Existing Predictor

The performance of SDM6A was compared with those of i6mA-Pred and iDNA6mA using an independent dataset. ACC, MCC, SN, and SP values showed that SDM6A comprehensively outperformed i6mA-Pred and iDNA6mA by more than 3.1%–5.8%, 6.3%–11.8%, 0.5%–5.9%, and 5.9%, respectively (Table 3). It is generally assumed that DL methods perform better than do other ML-based algorithms,[32] which has been widely applied in protein structure and function prediction.[33–39] However, SMD6A consistently outperformed the DL-based method, iDNA6mA, on both benchmark and independent datasets, further emphasizing that systematic selection of feature encodings and two-layer ensemble models are essential for improved prediction. Furthermore, McNemar's chi-square test was used to determine whether the differences between SDM6A and existing predictors were statistically significant. At a p value threshold of 0.05, SDM6A significantly outperformed the other two methods. Notably, i6mA-Pred and iDNA6mA provide only class labels, without offering a detailed probability score, which is an important attribute for users. However, SDM6A provides both class label and probability score, demonstrating the advantage of this method over other predictive approaches.

The improved performance, shown by SDM6A, may be explained as follows: (1) because previous feature extraction methods were relatively simple, we systematically and comprehensively explored different types of feature encodings and determined that five feature encodings significantly contribute to prediction of 6mA sites; (2) we optimized each feature encoding and individually integrated them via an ensemble strategy for SVM and ERT; and (3) we developed an ensemble model by integrating SVM and ERT, which further improved robustness of the model.

Web Server Implementation

To maximize the convenience for the users, we implemented a user-friendly and publicly accessible web server to predict novel putative 6mA sites in the rice genome. SDM6A is freely accessible at http://thegleelab.org/SDM6A. All datasets, utilized in this study, can be freely downloaded from our web server. The instructions of SDM6A usage has been provided in the following link: http://thegleelab.org/SDM6A/SDM6Atutorial.html.

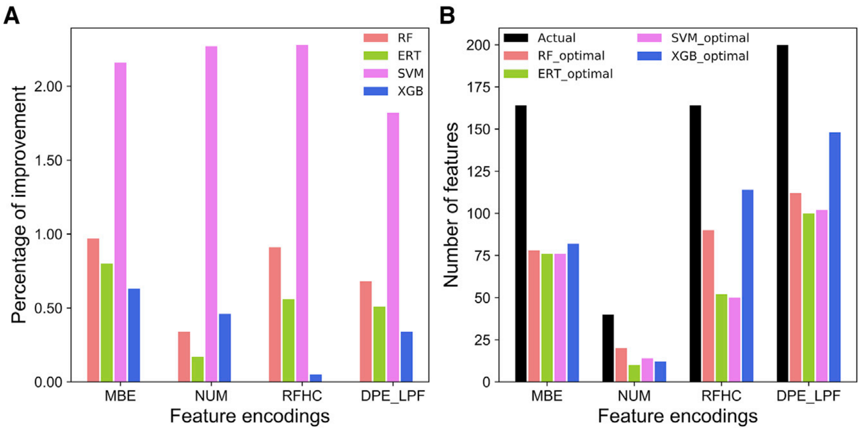

Figure 5. Comparison of Original Features and Optimal Features in Terms of Performance and Feature Dimension

(A) Percentage of improvement for each optimal feature-set encoding with respect to four different classifiers. (B) Comparison of original feature and optimal feature dimension for each feature encoding with respect to four different classifiers.

CONCLUSIONS

Identification of 6mA sites is essential for understanding epigenetic modifications occurring in the genome. Few computational methods have been developed for *in silico* prediction of 6mA sites.[18,19] Currently, there are no studies conducting a systematic and comprehensive analysis of informative features, effectiveness, and potential integration of ML methods. In this study, we developed a novel computational predictor called SDM6A. To generate a robust prediction model, we first used systematic and comprehensive analysis of various feature encodings, which revealed that five encoding methods were suitable for identifying 6mA sites. Optimal features were then selected for four encodings (BPF, DPE_LPF, NUM, and RFHC), and one encoding (KNN) was used because of small feature dimension. Corresponding models were developed separately for SVM and ERT. The one-layer ensemble model was constructed by averaging the prediction outputs of five different feature encodings individually for SVM and ERT. Subsequently, a second-layer ensemble model was constructed by averaging the prediction outputs of SVM and ERT, which improved robustness of the model.

In comparing the performance of SDM6A with those of state-of-the-art predictors (i6mA-Pred and iDNA6mA) using both benchmark and independent datasets revealed that SDM6A achieved the best performance with both datasets. This result shows that SDM6A was indeed more effective than state-of-the-art predictors in distinguishing 6mA sites from non-6mA sites. A user-friendly web server, based on the optimal ensemble model, was developed for use by the research community. In summary, complementary and heterogeneous features can help improve predictor performance.[40–42] Therefore, we will explore other informative features and increasing training dataset based on the experimental data availability in the future, which may help to develop next generation prediction model. The computational framework proposed in this work will assist in studies examining 6mA sites and other important epigenetic modifications such as 4mC and 5mC sites.[19,27,43,44] The current approach can be used in computational biology to develop other novel methods and can be widely applied to predict 6mA sites and to inspire development of next-generation predictors.

MATERIALS AND METHODS

Data Collection and Pre-processing

Constructing a high-quality dataset is essential for developing a reliable prediction model. In this study, we used the high-quality benchmark dataset generated by Chen et al.[18] for development or training of a prediction model. A benchmark dataset comprises 880 6mA (positive) and 880 non-6mA (negative) samples, with each sample possessing a central adenine NT having a length of 41 base pairs. Each positive sample is experimentally verified using an associated modification score (ModQV). If the ModQV score is above 30, it indicates that the related adenine NT is modified. Because there are no experimentally validated negative samples, Chen et al.[18] constructed a negative dataset using coding sequences containing GAGG motifs based on the findings of Zhou et al.,[17] who showed frequent 6mA modifications at GAGG motifs and less enrichment at the coding sequences. Importantly, the benchmark dataset is nonredundant, and sequence identity in negative or positive samples is reduced to less than 60% using CD-HIT.[45]

To evaluate the prediction model developed in this study, we constructed an independent dataset using the procedure employed by Chen et al.[18] The 6mA sites were downloaded from (https://www.ncbi.nlm.nih.gov/geo/query/acc.cgi?acc=GSE103145), and samples with ModQV score below 30, as well as those sharing >60% sequence identity with benchmark positive and negative datasets, were excluded. Finally, 221 6mA sequences were obtained and supplemented with an equal number of negative samples acquired from coding sequences that contained GAGG motifs, an adenine at the center, and were not detected via SMRT-seq. Notably, none of these positive and negative samples shared sequence identity of greater than 60% within independent and benchmark datasets, thereby excluding the possibility of overestimating predictive performance introduced by sequence identities.

Feature Extraction

Feature extraction, which directly impacts both accuracy and efficiency, is one of the most important steps in the development of ML-based models. In this study, extracted features were categorized into three groups: (1) sequence-based features, (2) physicochemical-based features, and (3) evolutionary-derived features.

Table 1. Performance Comparison of Different Single Method-Based Models and a Selection of Ensemble Models for Predicting 6mA Sites on the Benchmarking Dataset

Method	MCC	ACC	SN	SP	AUC
1. RF	0.759	0.878	0.840	0.917	0.942
2.ERT	0.759	0.878	0.844	0.913	0.946
3. SVM	0.751	0.875	0.852	0.898	0.935
4. XGB	0.748	0.874	0.860	0.889	0.947
{1, 2}	0.760	0.880	0.872	0.889	0.945
{2, 3}[a]	0.763	0.881	0.852	0.909	0.940
{3, 4}	0.757	0.878	0.874	0.883	0.944
{1, 3}	0.753	0.877	0.870	0.883	0.939
{1, 4}	0.756	0.878	0.873	0.883	0.947
{2, 4}	0.758	0.879	0.875	0.883	0.948
{1, 2, 3}	0.760	0.880	0.860	0.899	0.942
{2, 3, 4}	0.758	0.879	0.875	0.883	0.945
{1, 3, 4}	0.757	0.878	0.859	0.898	0.944
{1, 2, 4}	0.758	0.879	0.874	0.884	0.947
{1, 2, 3, 4}	0.756	0.878	0.865	0.891	0.945
i6mA-Pred[b]	0.670	0.835	0.834	0.836	0.909
iDNA6mA[b]	0.732	0.867	0.866	0.866	0.931

The first column represents a single method-based model or an ensemble model, which was built based on combining different single models. For instance, "1. RF" stands for the prediction model developed on RF, while "{1, 2}" means for ensemble model that is built based on single models numbered "1" and "2." Abbreviations are as follows: MCC, Matthews correlation coefficient; ACC, accuracy; SN, sensitivity; SP, specificity; and AUC, area under the curve.

[a]The optimal model was selected by systematically examining all possible random combinations.

[b]The existing method used for the comparison, whose metrics are taken from the corresponding references.[18,19]

Sequence-Derived Features

1. Numerical Representation of Nucleotides. Xu et al.[46] and Zhang et al.[40] have recently proposed a feature called numerical representation of amino acids, which has been successfully used to predict post-translational modifications. Based on these previous findings, numerical representation of amino acids was modified accordingly for NTs. NUM converts NT sequences into sequences of numerical values by mapping NTs in an alphabetical order. The four standard NTs, namely A, C, G, and T, are represented as 0.25, 0.50, 0.75, and 1.0, respectively; the length of each NT is 41, with 20 NTs upstream, a central adenine, and 20 NTs downstream. The central adenine, however, is ignored during calculations; only the upstream and downstream NTs are considered, thereby generating a 40-dimensional vector.

2. Mononucleotide Binary Encoding (MBE). The MBE method provides NT position-specific information,[22,47] where each NT is represented as a 4-dimensional binary vector of 0/1. For example, A, C, G, and T are respectively encoded with a binary vector of (1, 0, 0, 0), (0, 1, 0, 0), (0, 0, 1, 0), and (0, 0, 0, 1). In this study, a 164-dimensional vector was obtained for a given sequence length of 41 NTs.

3. DBE_LPF. This method involves two parts: (1) dinucleotide binary encoding (DBE) and (2) local position-specific dinucleotide frequency (LPF), which has been successfully used to predict N4-methylcytosine sites in DNA sequences[23] and N6-methyladenosine sites in RNA sequences.[48] DBE provides dinucleotide positional information, with each type of dinucleotide represented by a 4-dimensional vector of 0/1. For example, AA, AT, and AC are respectively encoded as (0, 0, 0, 0), (0, 0, 0, 1), and (0, 0, 1, 0). In this study, we obtained a 160-dimensional vector for a given sequence (41 NTs) containing 40 dinucleotides. LPF can be computed as $f = 1/|M_j| \, C(Y_{j-1}Y_j), 2 \leq j \leq K$, where K is the given sequence length, $|M_j|$ is the length of the jth prefix string $\{Y_1 Y_2 \ldots Y_j\}$ in the sequence, and $C(Y_{j-1}Y_j)$ is the frequency of the dinucleotide $Y_{j-1}Y_j$ in position j of the jth prefix string. A total of 200 features can be encoded per given sequence.

Physicochemical Features

Ring-Function-Hydrogen-Chemical Properties

Standard NTs have different chemical properties including rings, functional groups, and hydrogen bonds. These properties are grouped as follows: (1) (A, G) and (C, T), respectively, contain one and two rings; (2) (A, T) and (C, G), respectively, contain two and three hydrogen bonds; and (3) (A, C) and (G, T), respectively, contain amino and keto groups.[22,47,49,50] To include these properties, a given DNA sequence, encoded as a 4-dimensional vector (a, b, c, d_i), can be computed as follows:

$$a_i = \begin{cases} 1, if \ S_i \in \{A, G\} \\ 0 \ if \ S_i \in \{T, C\} \end{cases}, b = \begin{cases} 1, if \ S_i \in \{A, T\} \\ 0 \ if \ S_i \in \{C, G\} \end{cases}, c_i = \begin{cases} 1, if \ S_i \in \{A, C\} \\ 0 \ if \ S_i \in \{T, G\} \end{cases}, \quad (1)$$

where A, C, G, and T are represented by the coordinates (1, 1, 1), (0, 0, 1), (1, 0, 0), and (0, 1, 0), respectively. The density (d_i) of the NT (N_i) in a given sequence can be computed as follows:

$$d_i = \frac{1}{|M_i|} \sum_{j=1}^{K} f(n_j), f(n_j) = \begin{cases} 1, & if \ n_j = q \in \{A, T, G, C\} \\ 0, & else \end{cases} \quad (2)$$

where $|M_i| \, |N_i|$ is the length from the current NT position to the first NT, and q is any one of the four standard NTs. By integrating NT chemical properties and composition (combining Equations 1 and 2), a 41-NT sequence is encoded as a 164 (4 × 41)-dimensional vector.

Evolutionarily Derived Features

K-Nearest Neighbor

KNN encoding generates features for a given sequence based on the similarity of that sequence to n samples from both positive and negative sets. For two local sequences P_1 and P_2, the similarity score $S(P_1, P_2)$ is formulated as:

$$S(P_1, P_2) = \sum_{i=1}^{L} score(P_1(i), P_2(i)) \quad (3)$$

where $P_1(i)$ and $P_2(i)$ represent NTs at the ith position of sequences P_1 and P_2, respectively, and L is the length of the segment. For two NTs a and b, the similarity score is defined as:

Table 2. Performance Comparison of Various Single Method-Based Models and a Selected Ensemble Model for Predicting m6A Sites on the Independent Dataset

Method	MCC	ACC	SN	SP	AUC
1. RF	0.715	0.858	0.842	0.873	0.923
2.ERT	0.729	0.864	0.855	0.873	0.934
3. SVM	0.742	0.871	0.873	0.869	0.936
4. XGB	0.721	0.860	0.891	0.828	0.939
{1, 2}	0.726	0.862	0.896	0.828	0.931
{2, 3}[a]	0.769	0.885	0.878	0.891	0.938
{3, 4}	0.757	0.878	0.905	0.851	0.940
{1, 3}	0.743	0.871	0.891	0.851	0.935
{1, 4}	0.731	0.864	0.905	0.824	0.936
{2, 4}	0.731	0.864	0.905	0.824	0.938
{1, 2, 3}	0.751	0.876	0.887	0.864	0.936
{2, 3, 4}	0.744	0.871	0.905	0.837	0.939
{1, 3, 4}	0.760	0.880	0.891	0.869	0.938
{1, 2, 4}	0.735	0.867	0.905	0.828	0.936
{1, 2, 3, 4}	0.731	0.864	0.905	0.824	0.936

The first column represents a single method-based model or an ensemble model, which was built based on combining different single models (see Table 1 legend for more information).
[a]The best performance obtained by the optimal model.

Similarity score

$$Sim(a,b) = \begin{cases} +2, if\ a=b; \\ -1,\ else \end{cases} \quad (4)$$

In this study, we used n with values of 2, 4, 8, 16, 32, 64, and 128 to generate a 7-dimensional vector for a given sequence.

Feature Optimization

Feature optimization, used to improve classification performance, is one of the important steps in ML.[51] In this study, an F-score algorithm with a sequential forward search (SFS) protocol was used to filter out noisy and irrelevant features, after which a subset containing optimal features was selected. This two-step protocol has been successfully applied in various predictions.[23,52,53] In the first step, an F-score algorithm is used to rank the actual features, and to sort these features in a descending order, thereby generating a ranked feature list. The F-score of the ith feature is defined as:

$$F-score(i) = \frac{\left(\bar{x}_i^{(+)} - \bar{x}_i\right)^2 + \left(\bar{x}_i^{(-)} - \bar{x}_i\right)^2}{\frac{1}{n^+ - 1}\sum_{j=1}^{n^+}\left(\bar{x}_{i,j}^{(+)} - \bar{x}_i^{(+)}\right)^2 + \frac{1}{n^- - 1}\sum_{j=1}^{n^-}\left(\bar{x}_{i,j}^{(-)} - \bar{x}_i^{(-)}\right)^2} \quad (5)$$

where $\bar{x}_i$, $\bar{x}_i^{(+)}$, and $\bar{x}_i^{(-)}$, represent mean values of the ith feature in the combined (both positive and negative), positive, and negative datasets, respectively. n^+ and n^- represent the number of positive and negative samples, respectively. $\bar{x}_{i,j}^{(+)}$ and $\bar{x}_{i,j}^{(-)}$ represent the ith feature of jth positive instance and i^{th} feature of jth negative instance, respectively.

Table 3. Performances of the Proposed Method and Two State-of-Art Predictors on Independent Dataset

Method	MCC	ACC	SN	SP	AUC	p Value
SDM6A	0.765	0.882	0.878	0.887	0.938	—
i6mA-Pred	0.647	0.824	0.819	0.828	NA	< 0.0001[a]
iDNA6mA	0.702	0.851	0.873	0.828	NA	< 0.0001[a]

The first column represents the method evaluated in this study. Because i6mA-Pred and iDNA6mA did not provide predicted probability value, AUC value cannot be computed.
[a]A p value < 0.001 was considered to indicate a statistically significant difference between SDM6A and the selected method.

In the second step, two features were chosen from the ranked features list, and added sequentially as an input feature to four different ML classifiers (SVM, ERT, RF, and XGB); this was used for training and developing the corresponding prediction models. Ultimately, the features corresponding to the model with highest accuracy were recognized as optimal features for the respective ML classifier.

Machine Learning Algorithms

In this study, four different ML classifiers, namely SVM, ERT, RF, and XGB, were explored. Among these four algorithms, SD6MA integrated only two classifiers. The parameter search ranges and implementation used for the remaining two methods (RF and XGB) were similar to those utilized in previous studies.[54–58] Python packages, scikit-learn (version 0.18.1)[59] and xgboost[60] were implemented for all four classifiers.

Support Vector Machine

SVM, which has been extensively used in the fields of bioinformatics and computational biology, is one of the most powerful ML algorithms.[18,21,42,54,61–72] The objective of SVM is to find an optimal hyperplane that can maximize the distance between positive and negative samples in a high-dimensional feature space.[73] We implemented the radial basis function $K(x_i, x_j) = \exp(-\gamma|x_i - x_j|^2)$ as the Kernel function. Regularization parameters, such as penalty parameter C and kernel parameter γ of the SVM algorithm, were optimized using a grid search approach. The search ranges for the two parameters are $2^{-5} \le C \le 2^{15}$ with a step size of 2, and $2^{-15} \le \gamma \le 2^{-5}$ with a step size of 2^{-1}, respectively.

Extremely Randomized Tree

ERT, another powerful ML method developed by Geurts et al.,[74] has been widely used in various sequence-based prediction scenarios.[41,75] ERT is designed to reduce the variance of the model by incorporating a stronger randomization method. The ERT algorithm is similar to that of RF, except for two main differences: (1) ERT does not perform

a bagging procedure, but instead uses all training samples to construct each tree with varying parameters; and (2) rather than the best split used in RF, ERT randomly chooses the node split upon construction of each tree. The grid search approach is used for optimizing the number of trees (*ntree*), number of randomly selected features (*mtry*), and minimum number of samples required to split an internal node (*nsplit*) of the ERT algorithm. The search ranges for the three parameters were $50 \leq \text{ntree} \leq 2{,}000$ with a step size of 25, $1 \leq \text{m try} \leq 15$ with a step size of 1, and $1 \leq \text{nsplit} \leq 12$ with a step size of 1, respectively.

Cross-Validation

In statistical analysis method, K-fold CV has been widely used to evaluate the performance of ML classifiers. In this study, a 10-fold CV test was performed to evaluate model performance. In 10-fold CV, the benchmark dataset was randomly divided into 10 exclusive subsets of approximately equal size, with each subset containing an equal number of positive and negative samples. At each validation step, a single subset was retained as the validation set for evaluating model performance; the remaining nine subsets were used as training sets. This procedure was repeated 10 times, until each subset was used at least once as a validation set. Model performances on the 10 test subsets were then averaged, providing an estimate of the overall performance of the model on a 10-fold CV test.

Performance Assessment

Four sets of metrics, commonly used in the fields of computational biology and bioinformatics, were utilized to quantitatively evaluate the performance of the proposed method.[76–78] These metrics included sensitivity SN, SP, ACC, and MCC, and were computed as follows:

$$\begin{cases} SN = \dfrac{TP}{TP + FN} \\ SP = \dfrac{TN}{TN + FP} \\ ACC = \dfrac{TP + TN}{TP + TN + FN + FP} \\ MCC = \dfrac{TP * TN - FP * FN}{\sqrt{(TP + FN)(TP + FP)(TN + FP)(TN + FN)}} \end{cases} \quad (6)$$

Where TP is the number of 6mA samples correctly classified in prediction, and TN represents the number of non-6mA samples correctly classified by predictors. FP and FN represent the numbers of 6mA or non-6mA samples misclassified, respectively. Receiver-operating characteristic (ROC) curve and area under ROC curve (AUC) were used to assess overall performance. The closeness of the ROC curve to the left corner determines the closeness of AUC value to 1, which suggests better overall performance.

SUPPLEMENTAL INFORMATION

Supplemental Information can be found online at https://doi.org/10.1016/j.omtn.2019.08.011.

AUTHOR CONTRIBUTIONS

S.B., B.M., and G.L. conceived the project and designed the experiments. B.M., S.B., and T.H.S. performed the experiments and analyzed the data. S.B, B.M., and G.L. wrote the manuscript. All authors read and approved the final manuscript.

CONFLICTS OF INTEREST

The authors declare no competing interests.

ACKNOWLEDGMENTS

This work has been supported by the Basic Science Research Program through the National Research Foundation (NRF) of Korea funded by the Korea government (grant numbers 2018R1D1A1B07049572 and 2019R111A1A01062260), ICT and Future Planning (grant number 2016M3C7A1904392), and by the Korea Basic Science Institute (KBSI) National Research Facilities & Equipment Center (NFEC) grant funded by the Korea government (Ministry of Education) (grant number 2019R1A6C1010003).

REFERENCES

1. Shi, D.Q., Ali, I., Tang, J., and Yang, W.C. (2017). New Insights into 5hmC DNA Modification: Generation, Distribution and Function. Front. Genet. *8*, 100.
2. Liang, Z., Shen, L., Cui, X., Bao, S., Geng, Y., Yu, G., Liang, F., Xie, S., Lu, T., Gu, X., et al. (2018). DNA N(6)-Adenine Methylation in Arabidopsis thaliana. Dev. Cell *45*, 406–416 e403.
3. Feng, S., Cokus, S.J., Zhang, X., Chen, P.Y., Bostick, M., Goll, M.G., Hetzel, J., Jain, J., Strauss, S.H., Halpern, M.E., et al. (2010). Conservation and divergence of methylation patterning in plants and animals. Proc. Natl. Acad. Sci. USA *107*, 8689–8694.
4. Fu, Y., Luo, G.Z., Chen, K., Deng, X., Yu, M., Han, D., Hao, Z., Liu, J., Lu, X., Dore, L.C., et al. (2015). N6-methyldeoxyadenosine marks active transcription start sites in Chlamydomonas. Cell *161*, 879–892.
5. Greer, E.L., Blanco, M.A., Gu, L., Sendinc, E., Liu, J., Aristizábal-Corrales, D., Hsu, C.H., Aravind, L., He, C., and Shi, Y. (2015). DNA Methylation on N6-Adenine in C. elegans. Cell *161*, 868–878.
6. Zhang, G., Huang, H., Liu, D., Cheng, Y., Liu, X., Zhang, W., Yin, R., Zhang, D., Zhang, P., Liu, J., et al. (2015). N6-methyladenine DNA modification in Drosophila. Cell *161*, 893–906.
7. Koziol, M.J., Bradshaw, C.R., Allen, G.E., Costa, A.S.H., Frezza, C., and Gurdon, J.B. (2016). Identification of methylated deoxyadenosines in vertebrates reveals diversity in DNA modifications. Nat. Struct. Mol. Biol. *23*, 24–30.
8. Liu, J., Zhu, Y., Luo, G.Z., Wang, X., Yue, Y., Wang, X., Zong, X., Chen, K., Yin, H., Fu, Y., et al. (2016). Abundant DNA 6mA methylation during early embryogenesis of zebrafish and pig. Nat. Commun. *7*, 13052.
9. Wu, T.P., Wang, T., Seetin, M.G., Lai, Y., Zhu, S., Lin, K., Liu, Y., Byrum, S.D., Mackintosh, S.G., Zhong, M., et al. (2016). DNA methylation on N(6)-adenine in mammalian embryonic stem cells. Nature *532*, 329–333.
10. Wang, Y., Chen, X., Sheng, Y., Liu, Y., and Gao, S. (2017). N6-adenine DNA methylation is associated with the linker DNA of H2A.Z-containing well-positioned nucleosomes in Pol II-transcribed genes in Tetrahymena. Nucleic Acids Res. *45*, 11594–11606.
11. Luo, G.Z., Blanco, M.A., Greer, E.L., He, C., and Shi, Y. (2015). DNA N(6)-methyladenine: a new epigenetic mark in eukaryotes? Nat. Rev. Mol. Cell Biol. *16*, 705–710.
12. Vanyushin, B.F., Tkacheva, S.G., and Belozersky, A.N. (1970). Rare bases in animal DNA. Nature *225*, 948–949.
13. Smith, Z.D., and Meissner, A. (2013). DNA methylation: roles in mammalian development. Nat. Rev. Genet. *14*, 204–220.

14. Luo, G.Z., Wang, F., Weng, X., Chen, K., Hao, Z., Yu, M., Deng, X., Liu, J., and He, C. (2016). Characterization of eukaryotic DNA N(6)-methyladenine by a highly sensitive restriction enzyme-assisted sequencing. Nat. Commun. *7*, 11301.

15. Fang, G., Munera, D., Friedman, D.I., Mandlik, A., Chao, M.C., Banerjee, O., Feng, Z., Losic, B., Mahajan, M.C., Jabado, O.J., et al. (2012). Genome-wide mapping of methylated adenine residues in pathogenic Escherichia coli using single-molecule real-time sequencing. Nat. Biotechnol. *30*, 1232–1239.

16. Krais, A.M., Cornelius, M.G., and Schmeiser, H.H. (2010). Genomic N(6)-methyladenine determination by MEKC with LIF. Electrophoresis *31*, 3548–3551.

17. Zhou, C., Wang, C., Liu, H., Zhou, Q., Liu, Q., Guo, Y., Peng, T., Song, J., Zhang, J., Chen, L., et al. (2018). Identification and analysis of adenine N^6-methylation sites in the rice genome. Nat. Plants *4*, 554–563.

18. Chen, W., Lv, H., Nie, F., and Lin, H. (2019). i6mA-Pred: Identifying DNA N6-methyladenine sites in the rice genome. Bioinformatics *35*, 2796–2800.

19. Tahir, M., Tayara, H., and Chong, K.T. (2019). iDNA6mA (5-step rule): Identification of DNA N6-methyladenine sites in the rice genome by intelligent computational model via Chou's 5-step rule. Chemometr. Intell. Lab. Syst. *189*, 96–101.

20. Le, N.Q.K. (2019). iN6-methylat (5-step): identifying DNA N6-methyladenine sites in rice genome using continuous bag of nucleobases via Chou's 5-step rule. Mol. Genet. Genomics. Published online May 4, 2019. https://doi.org/10.1007/s00438-019-01570-y.

21. Manavalan, B., Basith, S., Shin, T.H., Wei, L., and Lee, G. (2019). Meta-4mCpred: A Sequence-Based Meta-Predictor for Accurate DNA N4-methylcytosine Site Prediction Using Effective Feature Representation. Mol. Ther. Nucleic Acids *16*, 733–744.

22. Wei, L., Su, R., Luan, S., Liao, Z., Manavalan, B., Zou, Q., et al. (2019). Iterative feature representations improve N4-methylcytosine site prediction. Bioinformatics. Published online May 17, 2019. https://doi.org/10.1093/bioinformatics/btz408.

23. Wei, L., Luan, S., Nagai, L.A.E., Su, R., and Zou, Q. (2018). Exploring sequence-based features for the improved prediction of DNA N4-methylcytosine sites in multiple species. Bioinformatics *15*, 1326–1333.

24. Wang, J., Li, J., Yang, B., Xie, R., Marquez-Lago, T.T., Leier, A., et al. (2018). Bastion3: a two-layer ensemble predictor of type III secreted effectors. Bioinformatics *35*, 2017–2028.

25. Wang, J., Yang, B., An, Y., Marquez-Lago, T., Leier, A., Wilksch, J., et al. (2019). Systematic analysis and prediction of type IV secreted effector proteins by machine learning approaches. Brief. Bioinform *20*, 931–951, 29186295Brief. Bioinform.

26. Wang, J., Yang, B., Leier, A., Marquez-Lago, T.T., Hayashida, M., Rocker, A., Zhang, Y., Akutsu, T., Chou, K.C., Strugnell, R.A., et al. (2018). Bastion6: a bioinformatics approach for accurate prediction of type VI secreted effectors. Bioinformatics *34*, 2546–2555.

27. Chen, W., Yang, H., Feng, P., Ding, H., and Lin, H. (2017). iDNA4mC: identifying DNA N4-methylcytosine sites based on nucleotide chemical properties. Bioinformatics *33*, 3518–3523.

28. Lai, H.Y., Chen, X.X., Chen, W., Tang, H., and Lin, H. (2017). Sequence-based predictive modeling to identify cancerlectins. Oncotarget *8*, 28169–28175.

29. Shao, L., Gao, H., Liu, Z., Feng, J., Tang, L., and Lin, H. (2018). Identification of Antioxidant Proteins With Deep Learning From Sequence Information. Front. Pharmacol. *9*, 1036.

30. Wei, L., Chen, H., and Su, R. (2018). M6APred-EL: A Sequence-Based Predictor for Identifying N6-methyladenosine Sites Using Ensemble Learning. Mol. Ther. Nucleic Acids *12*, 635–644.

31. Manavalan, B., Shin, T.H., and Lee, G. (2017). DHSpred: support-vector-machine-based human DNase I hypersensitive sites prediction using the optimal features selected by random forest. Oncotarget *9*, 1944–1956.

32. Xue, L., Tang, B., Chen, W., and Luo, J. (2018). DeepT3: deep convolutional neural networks accurately identify Gram-Negative Bacterial Type III Secreted Effectors using the N-terminal sequence. Bioinformatics *35*, 2051–2057.

33. Cao, R., Bhattacharya, D., Hou, J., and Cheng, J. (2016). DeepQA: improving the estimation of single protein model quality with deep belief networks. BMC Bioinformatics *17*, 495.

34. Cao, R., Freitas, C., Chan, L., Sun, M., Jiang, H., and Chen, Z. (2017). ProLanGO: Protein Function Prediction Using Neural Machine Translation Based on a Recurrent Neural Network. Molecules *22*, 22.

35. Conover, M., Staples, M., Si, D., Sun, M., and Cao, R. (2019). AngularQA: protein model quality assessment with LSTM networks. Computational and Mathematical Biophysics *7*, 1–9.

36. Hou, J., Wu, T., Cao, R., and Cheng, J. (2019). Protein tertiary structure modeling driven by deep learning and contact distance prediction in CASP13. Proteins. Published online February 17, 2019. https://doi.org/10.1101/552422.

37. Moritz, S., Pfab, J., Wu, T., Hou, J., Cheng, J., Cao, R., et al. (2019). Cascaded-CNN: deep learning to predict protein backbone structure from high-resolution cryo-EM density maps. bioRxiv. Published online March 12, 2019. https://doi.org/10.1101/572990.

38. Staples, M., Chan, L., Si, D., Johnson, K., Whyte, C., and Cao, R. (2019). Artificial Intelligence for Bioinformatics: Applications in Protein Folding Prediction. bioRxiv. Published online February 28, 2019. https://doi.org/10.1101/561027.

39. Stephenson, N., Shane, E., Chase, J., Rowland, J., Ries, D., Justice, N., Zhang, J., Chan, L., and Cao, R. (2019). Survey of Machine Learning Techniques in Drug Discovery. Curr. Drug Metab. *20*, 185–193.

40. Zhang, Y., Xie, R., Wang, J., Leier, A., Marquez-Lago, T.T., Akutsu, T., Webb, G.I., Chou, K.C., and Song, J. (2018). Computational analysis and prediction of lysine malonylation sites by exploiting informative features in an integrative machine-learning framework. Brief. Bioinform. Published online August 24, 2018. https://doi.org/10.1093/bib/bby079.

41. Basith, S., Manavalan, B., Shin, T.H., and Lee, G. (2018). iGHBP: Computational identification of growth hormone binding proteins from sequences using extremely randomised tree. Comput. Struct. Biotechnol. J. *16*, 412–420.

42. Manavalan, B., Basith, S., Shin, T.H., Wei, L., and Lee, G. (2019). mAHTPred: a sequence-based meta-predictor for improving the prediction of anti-hypertensive peptides using effective feature representation. Bioinformatics *35*, 2757–2765.

43. Pavlovic, M., Ray, P., Pavlovic, K., Kotamarti, A., Chen, M., and Zhang, M.Q. (2017). DIRECTION: a machine learning framework for predicting and characterizing DNA methylation and hydroxymethylation in mammalian genomes. Bioinformatics *33*, 2986–2994.

44. Zhang, M., Xu, Y., Li, L., Liu, Z., Yang, X., and Yu, D.J. (2018). Accurate RNA 5-methylcytosine site prediction based on heuristic physical-chemical properties reduction and classifier ensemble. Anal. Biochem. *550*, 41–48.

45. Fu, L., Niu, B., Zhu, Z., Wu, S., and Li, W. (2012). CD-HIT: accelerated for clustering the next-generation sequencing data. Bioinformatics *28*, 3150–3152.

46. Xu, Y., Ding, Y.X., Ding, J., Wu, L.Y., and Xue, Y. (2016). Mal-Lys: prediction of lysine malonylation sites in proteins integrated sequence-based features with mRMR feature selection. Sci. Rep. *6*, 38318.

47. Manavalan, B., Basith, S., Shin, T.H., Wei, L., and Lee, G. (2019). Meta-4mCpred: A Sequence-Based Meta-Predictor for Accurate DNA 4mC Site Prediction Using Effective Feature Representation. Mol. Ther. Nucleic Acids *16*, 733–744.

48. Qiang, X., Chen, H., Ye, X., Su, R., and Wei, L. (2018). M6AMRFS: Robust Prediction of N6-Methyladenosine Sites With Sequence-Based Features in Multiple Species. Front. Genet. *9*, 495.

49. Chen, W., Feng, P., Ding, H., Lin, H., and Chou, K.C. (2015). iRNA-Methyl: Identifying N(6)-methyladenosine sites using pseudo nucleotide composition. Anal. Biochem. *490*, 26–33.

50. Chen, W., Ding, H., Zhou, X., Lin, H., and Chou, K.C. (2018). iRNA(m6A)-PseDNC: Identifying N^6-methyladenosine sites using pseudo dinucleotide composition. Anal. Biochem. *561-562*, 59–65.

51. Wei, L., Zhou, C., Su, R., and Zou, Q. (2019). PEPred-Suite: improved and robust prediction of therapeutic peptides using adaptive feature representation learning. Bioinformatics. Published online April 17, 2019. https://doi.org/10.1093/bioinformatics/btz246.

52. Tan, J.-X., Dao, F.-Y., Lv, H., Feng, P.-M., and Ding, H. (2018). Identifying Phage Virion Proteins by Using Two-Step Feature Selection Methods. Molecules *23*, 2000.

53. Boopathi, V., Subramaniyam, S., Malik, A., Lee, G., Manavalan, B., and Yang, D.C. (2019). mACPpred: A Support Vector Machine-Based Meta-Predictor for Identification of Anticancer Peptides. Int. J. Mol. Sci. *20*, 20.

54. Manavalan, B., Basith, S., Shin, T.H., Choi, S., Kim, M.O., and Lee, G. (2017). MLACP: machine-learning-based prediction of anticancer peptides. Oncotarget *8*, 77121–77136.

55. Manavalan, B., Shin, T.H., Kim, M.O., and Lee, G. (2018). PIP-EL: A New Ensemble Learning Method for Improved Proinflammatory Peptide Predictions. Front. Immunol. *9*, 1783.

56. Yu, J., Shi, S., Zhang, F., Chen, G., and Cao, M. (2019). PredGly: Predicting lysine glycation sites for Homo sapiens based on XGboost feature optimization. Bioinformatics *35*, 2749–2756.

57. Manavalan, B., Shin, T.H., Kim, M.O., and Lee, G. (2018). AIPpred: Sequence-Based Prediction of Anti-inflammatory Peptides Using Random Forest. Front. Pharmacol. *9*, 276.

58. Manavalan, B., Subramaniyam, S., Shin, T.H., Kim, M.O., and Lee, G. (2018). Machine-Learning-Based Prediction of Cell-Penetrating Peptides and Their Uptake Efficiency with Improved Accuracy. J. Proteome Res. *17*, 2715–2726.

59. Abraham, A., Pedregosa, F., Eickenberg, M., Gervais, P., Mueller, A., Kossaifi, J., Gramfort, A., Thirion, B., and Varoquaux, G. (2014). Machine learning for neuroimaging with scikit-learn. Front. Neuroinform. *8*, 14.

60. Chen, T., and Guestrin, C. (2016). XGBoost: A Scalable Tree Boosting System. In Proceedings of the 22nd ACM SIGKDD International Conference on Knowledge Discovery and Data Mining (ACM), pp. 785–794.

61. Manavalan, B., and Lee, J. (2017). SVMQA: support-vector-machine-based protein single-model quality assessment. Bioinformatics *33*, 2496–2503.

62. Manavalan, B., Shin, T.H., and Lee, G. (2018). PVP-SVM: Sequence-Based Prediction of Phage Virion Proteins Using a Support Vector Machine. Front. Microbiol. *9*, 476.

63. Dao, F.Y., Lv, H., Wang, F., Feng, C.Q., Ding, H., Chen, W., et al. (2019). Identify origin of replication in Saccharomyces cerevisiae using two-step feature selection technique. Bioinformatics *35*, 2075–2083.

64. Feng, C.Q., Zhang, Z.Y., Zhu, X.J., Lin, Y., Chen, W., Tang, H., and Lin, H. (2019). iTerm-PseKNC: a sequence-based tool for predicting bacterial transcriptional terminators. Bioinformatics *35*, 1469–1477.

65. Feng, P., Yang, H., Ding, H., Lin, H., Chen, W., and Chou, K.C. (2019). iDNA6mA-PseKNC: Identifying DNA N^6-methyladenosine sites by incorporating nucleotide physicochemical properties into PseKNC. Genomics *111*, 96–102.

66. Tang, H., Zhao, Y.W., Zou, P., Zhang, C.M., Chen, R., Huang, P., and Lin, H. (2018). HBPred: a tool to identify growth hormone-binding proteins. Int. J. Biol. Sci. *14*, 957–964.

67. Chen, W., Tang, H., Ye, J., Lin, H., and Chou, K.C. (2016). iRNA-PseU: Identifying RNA pseudouridine sites. Mol. Ther. Nucleic Acids *5*, e332.

68. Feng, P., Ding, H., Yang, H., Chen, W., Lin, H., and Chou, K.C. (2017). iRNA-PseColl: Identifying the Occurrence Sites of Different RNA Modifications by Incorporating Collective Effects of Nucleotides into PseKNC. Mol. Ther. Nucleic Acids *7*, 155–163.

69. Chen, W., Feng, P., Yang, H., Ding, H., Lin, H., and Chou, K.C. (2018). iRNA-3typeA: Identifying Three Types of Modification at RNA's Adenosine Sites. Mol. Ther. Nucleic Acids *11*, 468–474.

70. Su, Z.D., Huang, Y., Zhang, Z.Y., Zhao, Y.W., Wang, D., Chen, W., Chou, K.C., and Lin, H. (2018). iLoc-lncRNA: predict the subcellular location of lncRNAs by incorporating octamer composition into general PseKNC. Bioinformatics *34*, 4196–4204.

71. Yang, H., Qiu, W.R., Liu, G., Guo, F.B., Chen, W., Chou, K.C., and Lin, H. (2018). iRSpot-Pse6NC: Identifying recombination spots in *Saccharomyces cerevisiae* by incorporating hexamer composition into general PseKNC. Int. J. Biol. Sci. *14*, 883–891.

72. Xu, Z.C., Feng, P.M., Yang, H., Qiu, W.R., Chen, W., and Lin, H. (2019). iRNAD: a computational tool for identifying D modification sites in RNA sequence. Bioinformatics. Published online May 11, 2019. https://doi.org/10.1093/bioinformatics/btz358.

73. Cortes, C., and Vapnik, V. (1995). Support-Vector Networks. Mach. Learn. *20*, 273–297.

74. Geurts, P., Ernst, D., and Wehenkel, L. (2006). Extremely randomized trees. Mach. Learn. *63*, 3–42.

75. Manavalan, B., Govindaraj, R.G., Shin, T.H., Kim, M.O., and Lee, G. (2018). iBCE-EL: A New Ensemble Learning Framework for Improved Linear B-Cell Epitope Prediction. Front. Immunol. *9*, 1695.

76. Su, R., Hu, J., Zou, Q., Manavalan, B., and Wei, L. (2019). Empirical comparison and analysis of web-based cell-penetrating peptide prediction tools. Brief. Bioinform. January 10, 2019. https://doi.org/10.1093/bib/bby124.

77. Feng, P.M., Chen, W., Lin, H., and Chou, K.C. (2013). iHSP-PseRAAAC: Identifying the heat shock protein families using pseudo reduced amino acid alphabet composition. Anal. Biochem. *442*, 118–125.

78. Chen, W., Feng, P., Liu, T., and Jin, D. (2019). Recent Advances in Machine Learning Methods for Predicting Heat Shock Proteins. Curr. Drug Metab. *20*, 224–228.

OMTN, Volume *18*

Supplemental Information

SDM6A: A Web-Based Integrative Machine-Learning Framework for Predicting 6mA Sites in the Rice Genome

Shaherin Basith, Balachandran Manavalan, Tae Hwan Shin, and Gwang Lee

Figure S1. Performance of four different ML classifiers with respect to using five feature descriptors to distinguish between 6mA sites and non-6mA sites on independent dataset. (A) RF, (B) ERT, (C) XGB, and (D) SVM.

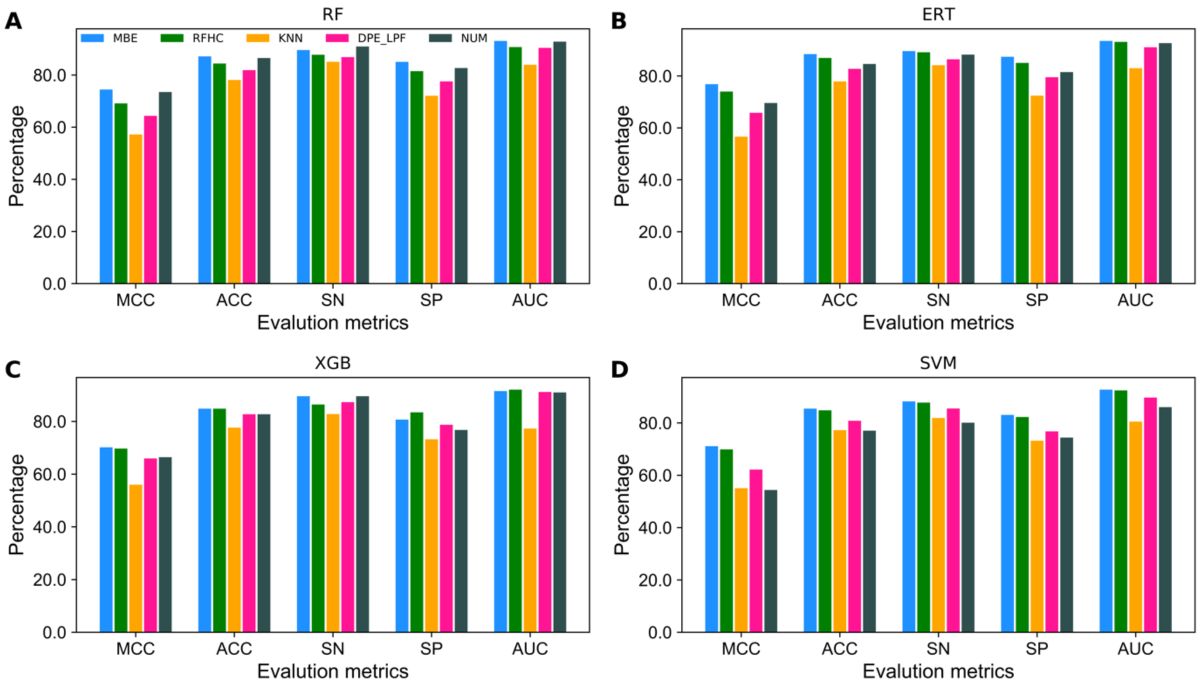

Figure S2. Performance of four different ML classifiers with respect to using nine feature descriptors to distinguish between 6mA sites and non-6mA sites on benchmark dataset. (A) RF, (B) ERT, (C) XGB, and (D) SVM.

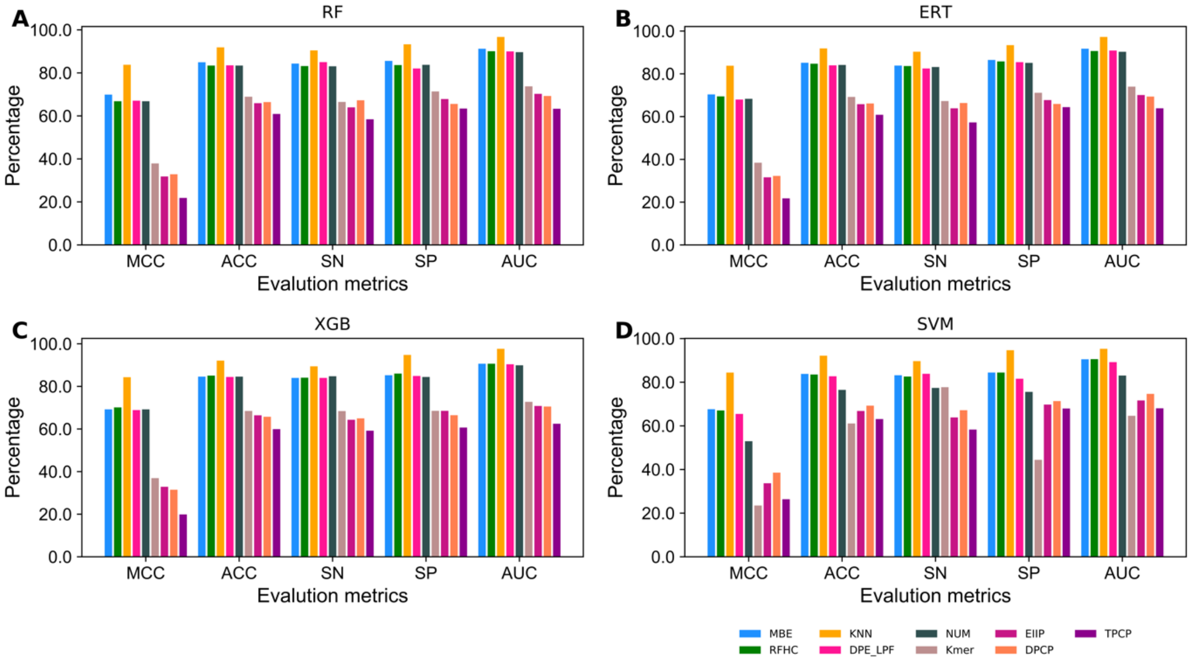

Table S1. Performances of four different classifiers using optimal feature set in distinguishing 6mAs and non-6mAs with respect to four different feature encodings.

Feature encoding	Method	MCC	ACC	SN	SP	AUC
MBE	RF	0.718	0.859	0.864	0.855	0.922
	ERT	0.719	0.860	0.858	0.861	0.925
	SVM	0.719	0.860	0.852	0.867	0.921
	XGB	0.705	0.852	0.867	0.838	0.921
NUM	RF	0.675	0.838	0.839	0.836	0.902
	ERT	0.686	0.843	0.836	0.850	0.910
	SVM	0.575	0.788	0.794	0.781	0.849
	XGB	0.701	0.851	0.854	0.847	0.914
RFHC	RF	0.687	0.843	0.831	0.856	0.909
	ERT	0.706	0.853	0.846	0.860	0.915
	SVM	0.717	0.858	0.838	0.878	0.917
	XGB	0.702	0.851	0.851	0.851	0.918
DPE_LPF	RF	0.684	0.842	0.843	0.841	0.912
	ERT	0.690	0.845	0.830	0.860	0.912
	SVM	0.691	0.846	0.850	0.841	0.911
	XGB	0.696	0.848	0.853	0.842	0.907

Table S2. Performance comparison between SDM6A and i6mA-Pred on benchmark dataset using jackknife test.

Method	MCC	ACC	SN	SP	AUC	P-value (AUC)
SDM6A	0.774	0.887	0.878	0.896	0.948	—
i6mA-Pred	0.660	0.831	0.830	0.831	0.886	<0.000001

First column represents the method name employed in this study. The second, third, fourth, and the fifth columns, respectively, represent the MCC, accuracy (ACC), sensitivity (SN), and specificity (SP). The sixth and the seventh columns, respectively, represent the AUC and pairwise comparison of ROC area under curves (AUCs) between SDM6A and the i6mA-Pred using a two-tailed *t*-test [1].

Reference

1. Hanley, JA, and McNeil, BJ (1982). The meaning and use of the area under a receiver operating characteristic (ROC) curve. *Radiology* **143**: 29-36.
